# Intra- and interspecific diversity analyses in the genus *Eremurus* in Iran using genotyping-by-sequencing reveal geographic population structure

**DOI:** 10.1038/s41438-020-0265-9

**Published:** 2020-03-02

**Authors:** Hanieh Hadizadeh, Bochra A. Bahri, Peng Qi, H. Dayton Wilde, Katrien M. Devos

**Affiliations:** 1 Department of Horticulture, Faculty of Agriculture, Tarbiat Modares University, Tehran, Iran; 2 Laboratory of Bioaggressors and Integrated Protection in Agriculture, The National Agronomic Institute of Tunisia, University of Carthage, 1082 Tunis, Tunisia; 3 Institute of Plant Breeding, Genetics and Genomics (Department of Crop and Soil Sciences), and Department of Plant Biology, University of Georgia, Athens, GA 30602 USA; 4 Institute of Plant Breeding, Genetics and Genomics (Department of Horticulture), University of Georgia, Athens, GA 30602 USA; 5 Present Address: Department of Plant Pathology, University of Georgia, Griffin, GA 30223 USA

**Keywords:** Genetic variation, Next-generation sequencing

## Abstract

*Eremurus* species, better known as ‘Foxtail Lily’ or ‘Desert Candle’, are important worldwide in landscaping and the cut-flower industry. One of the centers of highest diversity of the genus *Eremurus* is Iran, which has seven species. However, little is known about the genetic diversity within the genus *Eremurus*. With the advent of genotyping-by-sequencing (GBS), it is possible to develop and employ single nucleotide polymorphism (SNP) markers in a cost-efficient manner in any species, regardless of its ploidy level, genome size or availability of a reference genome. Population structure and phylogeographic analyses of the genus *Eremurus* in Iran using a minimum of 3002 SNP markers identified either at the genus level or at the species level from GBS data showed longitudinal geographic structuring at the country scale for the genus and for the species *E. spectabilis* and *E. luteus*, and at the regional scale for *E. olgae*. Our analyses furthermore showed a close genetic relatedness between *E. olgae* and *E. stenophyllus* to the extent that they should be considered subspecies within an *E. olgae/stenophyllus* species complex. Their close genetic relatedness may explain why crosses between these two (sub)species have been found in the wild and are exploited extensively as ornamentals. Last, current species identification, while robust, relies on flower morphology. A subset of seven SNPs with species-specific (private) alleles were selected that differentiate the seven *Eremurus* species. The markers will be especially useful for cultivar protection and in hybrid production, where true hybrids could be identified at the seedling stage.

## Introduction

*Eremurus*, the largest genus in the Asphodelaceae, is comprised of some 45 species of herbaceous perennial plants that are native to central Asia and Caucasia^1^. *Eremurus* species are important commercially as ornamental plants for landscaping and cut-flower markets^2^. Due to their large and colorful floral spikes, *Eremurus* species are known in the international horticulture trade as “Foxtail Lily” or “Desert Candle”. In addition to their ornamental value, *Eremurus* species have been used in traditional medicine and are potential sources for anti-inflammatory, antibacterial, and antiprotozoal drugs^3–5^. Other *Eremurus* products, such as bio-oil^6^ and adhesives^7^, have industrial applications.

Interspecific breeding of *Eremurus* species has been conducted for floral color and longevity, resulting in popular hybrids such as *Eremurus* × *isabellinus* (*E. stenophyllus* × *E. olgae*). A better understanding of the genetic variation within and among *Eremurus* species would facilitate breeding for ornamental traits and other properties. Naderi Safar and colleagues^8^ used genetic variation obtained by amplicon sequencing of the plastid *trnL-F* and nuclear rDNA ITS regions to conduct a molecular phylogenetic study of three Asphodelaceae genera, including *Eremurus*. This study showed that *Eremurus* species grouped into the paraphyletic subgenus *Henningia* and the monophyletic subgenus *Eremurus*. However, information on the genetic diversity within *Eremurus* species is lacking. Recent developments in next generation sequencing technologies have enabled the detection of single nucleotide polymorphism (SNP) markers at the whole genome level in non-model species, including those that lack a sequenced genome, using reduced representation sequencing^9–11^. These approaches have not yet been applied to identify SNP markers across species within an angiosperm genus comprised of species with very large genomes (>8 Gb) and no reference genome. Both diploid (*E. chinensis*) and tetraploid (*E. anisopterus*) *Eremurus* species have been identified by karyotype analysis with 2*n* chromosome counts of 14 and 28, respectively^12,13^. Flow cytometry of the diploid *E. stenophyllus* (2*n* = 2*x* = 14) determined that it has a large 2C genome size of 16.2 gigabases (1C = 8.1 Gb) and a GC content of 41.3%^14^.

Iran is the third largest diversity center of the genus *Eremurus*, after the Soviet Union and Afghanistan^15^. There are seven *Eremurus* species and three subspecies found in Iran, with the greatest species diversity located in the northeastern part of the country. *Eremurus stenophyllus* (Boiss. & Buhse) Baker subsp. *stenophyllus* is endemic to Iran and *E. kopet-daghensis* Karrer is subendemic^15^. *Eremurus stenophyllus* subsp. *stenophyllus* and *E. spectabilis* M. Bieb subsp. *subalbiflorus* are recognized as endangered and in need of conservation^16^. The other Iranian species/subspecies are *E. spectabilis* subsp. *spectabilis*, *E. persicus* (Jaub. & Spach) Boiss.*, E. olgae* Regel, *E. luteus* Baker, and *E. inderiensis* (M. Bieb.) Regel. Hybrids between *E. olgae* and *E. stenophyllus* subsp. *stenophyllus* have been observed in the wild and are identified as *E. x albocitrinus* Baker. *Eremurus* species are generally insect-pollinated, although self-fertilization is possible and wind dispersal of pollen has been observed in desert habitats where pollinator activity is unreliable^17,18^.

In this study, we investigated the interspecific and intraspecific diversity in *Eremurus spp*. germplasm from Iran using SNP markers identified through genotyping-by-sequencing (GBS)^10^ to determine phylogenetic relationships and investigate correlations between the genetic diversity, morphological diversity and geographic origin. In addition to the biological significance of our research, this is the first report of the use of GBS on species of the Asphodelaceae, none of which have been sequenced to date, the first use of GBS on an angiosperm species with a genome size (1C) larger than 8 Gb and no reference genome, and one of the few applications of GBS to plants of ornamental interest. Furthermore, we demonstrate the use of GBS to study intraspecific variation as well as interspecific variation in *Eremurus spp*. using different SNP-calling protocols on the same dataset.

## Results

### Genetic analyses across species within the genus *Eremurus*

#### SNP markers identified by GBS across *Eremurus* species

To analyze diversity in *Eremurus* at the genus level, a reference was assembled from GBS reads (‘GBS reference’) across 96 accessions belonging to seven *Eremurus* species collected across Iran (Supplementary Table S1; Supplementary Fig. S1). Because the reference building was carried out across species, we required each reference tag to be present in only two accessions in order to be included in the reference. The threshold we typically use for within species reference building is presence in at least 50% of the samples. The assembled GBS reference consisted of 201,099 tags. We obtained a total of 12,535 SNP markers across the 96 samples after alignment of the reads from each accession to the GBS reference and SNP calling, removal of adjacent SNPs (multiple side-by-side SNPs are sometimes caused by misalignment of reads) and filtering for biallelic SNPs, SNPs with a quality depth (QD) ≥ 10, and SNPs with <50% of missing data. Six accessions were removed from the analysis because they had <600,000 reads. An additional two samples with >1 M reads had >75% missing data and were also removed. The average number of reads for the remaining 88 accessions was 1.67 million (M), with minimum and maximum read numbers of 0.72 M and 11.43 M, respectively. We then decreased the missing data threshold for SNPs from 50 to 30%, and removed SNPs with a minor allele frequency ≤5%. The final number of SNPs used for the diversity analyses across the seven *Eremurus* species was 3002. A SNP resampling analysis showed that a subset of 1000 randomly selected SNPs had the same power to distinguish all multilocus genotypes as the full set of 3002 SNPs, indicating that our SNP set was adequate to determine the diversity between *Eremurus* species (Supplementary Fig. S2). The SNP markers, and the sequence of the corresponding GBS reads, are given in Supplementary Table S2. The genotypic scores for the 3002 SNP markers in each of the 88 accessions are given in Supplementary Table S3.

#### Population structure analysis

**Fig. 1:**
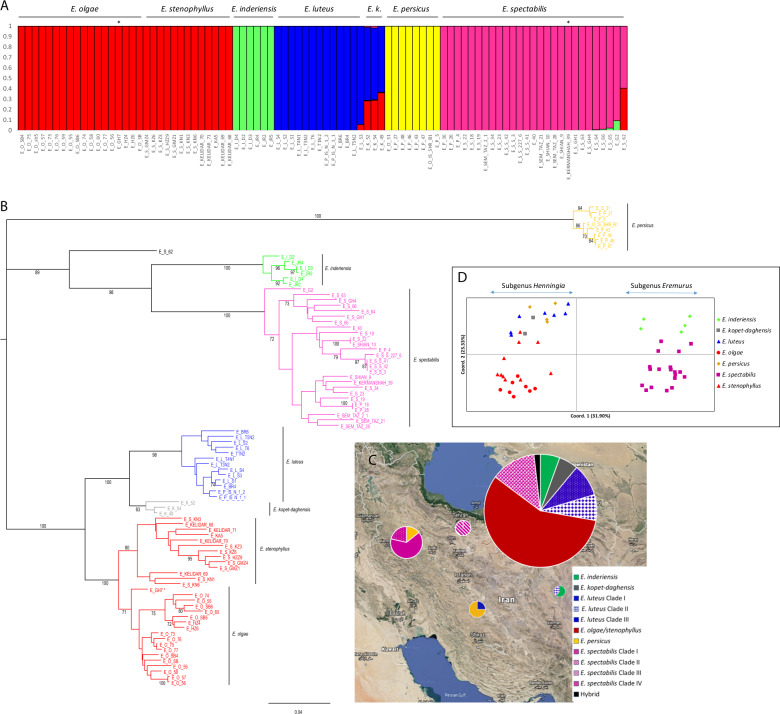
Genetic, morphological and geographic stratification of accessions belonging to seven species within genus *Eremurus*. **a** Genetic population groups as determined by STRUCTURE for *K* = 5. The subpopulations identified were *olgae/stenophyllus*, *inderiensis*, *luteus*, *persicus*, and *spectabilis*. Colored vertical bars indicate the percentage membership to different subpopulations. Genotypes indicated with * had not been morphologically classified at the species level. ‘*E. k.’* indicates *E. kopet-daghensis*. **b** NJ tree based on 3002 ‘species SNPs’ showing the relationships between seven *Eremurus* species. Bootstrap values for branches are indicated when higher than 70%. **c** Map showing the geographical distribution of the genetic groups of the *Eremurus* populations sampled across Iran. **d** Principal coordinates analysis (PCoA) using 16 morphological traits. Accessions are color-coded by species

The most likely number of subpopulations was determined by Structure Harvester^19^ (Delta *K* value) to be *K* = 5, after running STRUCTURE^20^ with *K* = 1 to *K* = 10 (Supplementary Fig. S3). The subpopulation division was largely by species (Fig. 1a). *Eremurus inderiensis*, *E. luteus*, *E. persicus*, and *E. spectabilis* each formed a single subpopulation, while *E. olgae* and *E. stenophyllus* grouped together. At *K* = 6 and when considering majority (≥ 50%) membership to a single subpopulation, all *E. olgae* accessions and two (25%) *E. stenophyllus* accessions formed one group, while the remaining *E. stenophyllus* accessions (75%) formed a second group (Supplementary Fig. S4). However, 39% of the *E. olgae* accessions and all of the *E. stenophyllus* accessions that belonged to the *‘olgae/stenophyllus’* subpopulation at *K* = 5 were admixed (≤ 90% membership to a single subpopulation) at *K* = 6. At *K* = 5, all three *E. kopet-daghensis* individuals were admixed with approximately 2/3 membership to subpopulation *‘luteus’* and 1/3 membership to subpopulation ‘*olgae/stenophyllus’* (Fig. 1a). The only other line that was admixed at *K* = 5 was E_S_62, which had 59% membership to subpopulation *‘spectabilis’* and 41% to ‘*olgae/stenophyllus’*. E_S_62, which had been identified morphologically as *E. spectabilis*, had a considerably higher number of heterozygous SNPs (31.1%) compared with the other *Eremurus* accessions (<10%).

#### Phylogeographic analyses within the genus *Eremurus*

Neighbor Joining (NJ) and Unweighted Pair Group Method with Arithmetic mean (UPGMA) analyses, carried out with the set of 3002 ‘species SNPs’, separated the seven species in very strongly supported clades (bootstrap values ≥98%) (Fig. 1b and Supplementary Fig. S5). The seven species’ clades were organized into three superclades. Interrelationships between the superclades were unresolved in the NJ tree (Fig. 1b), but the UPGMA tree topology suggested that superclades 1 and 2 were most closely related (Supplementary Fig. S5). Superclade 1 comprised *E. olgae*, *E. stenophyllus*, *E. luteus*, and *E. kopet-daghensis*. *E. olgae* was sister to *E. stenophyllus*, and *E. luteus* was sister to *E. kopet-daghensis*. Superclade 2 comprised two sister clades corresponding to *E. inderiensis* and *E. spectabilis*, and *E. persicus* formed superclade 3. The pairwise Nei’s genetic distances between the seven *Eremurus* species revealed *E. olgae* and *E. stenophylus* as the most closely related species (Nei = 0.059; Fst=0.217; Supplementary Table S4). With the exception of a few branches, relationships between accessions within species had low bootstrap values (Fig. 1b).

The genus *Eremurus* in Iran was geographically structured according to an East-West transect. Overall, a Mantel test revealed a significant correlation between genetic and geographic distances (Rxy = 0.439; *P* = 0.001). Within subgenus *Eremurus* (Supplementary Table S1), *E. spectabilis* was the dominant species in the western part of Iran, while *E. inderiensis* was only present in the eastern part of Iran (Fig. 1c). All species sampled within subgenus *Henningia* originated from the eastern part of Iran except *E. persicus*, which was only present in the west and center of Iran.

#### Private alleles that can be used for species identification

Overall, high genetic differentiation (Fst = 0.832) and a low level of gene flow (*N*_m_ = 0.579) were observed between the seven *Eremurus* species leading to the identification of a total of 864 private alleles (alleles that are unique to a single species and present in that species at a frequency of 100%) and 717 diagnostic alleles (alleles that are unique to a single species but present in that species at a frequency <100%) (Table 1). This represents 26.3% of all alleles. The SNP markers with private alleles (Supplementary Table S2) can be used to classify *Eremurus* at the species level. The highest number of private alleles (467) was found in *E. persicus*, the species with the highest Nei’s genetic distances from the other *Eremurus* species analyzed (Nei’s distances ≥0.626) (Supplementary Table S4). No private alleles were identified for *E. olgae* or *E. stenophyllus*, but both species did carry diagnostic alleles. The highest frequency of any diagnostic allele in *E. stenophyllus* was 88.5% while in *E. olgae*, the highest frequency was 61.8%. A total of 82 and 410 alleles were private and diagnostic, respectively, for the *E. olgae/E. stenophyllus* complex. The number of private and diagnostic alleles per subpopulation is given in Table 1. SNPs with private alleles are indicated in Supplementary Table S2.

**Table 1:** Summary of diversity indexes and private SNPs in *Eremurus* species using SNPs identified across all species (‘species SNPs’)

Species	SNP dataset	*N*	Ne (SE)	*I* (SE)	Ho (SE)	He (SE)	Fis (SE)	*P* (%)	Heterozygous SNPs (%)	Private SNPs	Diagnostic SNPs
*E. inderiensis*	3002	6	1.044 (0.006)	0.060 (0.003)	0.011 (0.001)	0.042 (0.002)	0.654 (0.010)	9.79	0.94	163	20
*E. kopet-daghensis*		3	0.969 (0.006)	0.033 (0.003)	0.009 (0.001)	0.023 (0.002)	0.586 (0.012)	5.16	0.71	18	–
*E. luteus*		13	1.079 (0.006)	0.088 (0.004)	0.023 (0.002)	0.060 (0.003)	0.509 (0.009)	16.89	2.08	106	146
*E. stenophyllus*		13	1.164 (0.006)	0.138 (0.005)	0.053 (0.002)	0.094 (0.003)	0.361 (0.009)	25.35	5.41	82 (0;0)^a^	410 (57;10)^a^
*E. olgae*		17	1.129 (0.005)	0.111 (0.004)	0.038 (0.002)	0.074 (0.003)	0.368 (0.009)	22.29	3.78		
*E. persicus*		8	0.880 (0.008)	0.039 (0.003)	0.008 (0.001)	0.027 (0.002)	0.557 (0.010)	7.26	0.70	467	54
*E. spectabilis*		26	1.124 (0.005)	0.106 (0.004)	0.030 (0.002)	0.071 (0.003)	0.450 (0.008)	22.52	2.94	28	87
Total		86	1.055 (0.002)	0.082 (0.001)	0.025 (0.001)	0.056 (0.001)	0.454 (0.004)	15.61	2.37	864	717

*N* number of accessions, *Ne* number of effective alleles, *I* Shannon’s information index, *Ho* observed heterozygosity, *He* expected heterozygosity, *Fis* fixation index, *SE* standard error, *P* percentage of polymorphic loci (%)

SNP dataset: number of SNPs in the dataset used to calculate the diversity indexes; heterozygous SNPs: percentage of heterozygous SNPs (%); private SNPs: number of SNPs fixed in one species (at a frequency of 100%) and absent in all other species; diagnostic SNPs: number of SNPs present in one species at a frequency below 100% and absent in all other species

E_S_62 and E_GH7 were not included in this analysis

^a^The two numbers in parentheses separated by a semi-colon correspond to the number of private/diagnostic SNPs recorded for *E. stenophyllus* (first number) and *E. olgae* (second number)

### Morphological analyses across species within the genus *Eremurus*

Tepal color was the most polymorphic trait evaluated across the *Eremurus* species with seven characters recorded and a Shannon diversity index *H*′ of 1.568. Rhizome diameter was the least powerful trait to differentiate the accessions morphologically with two characters recorded and an *H*′ of 0.251 (Supplementary Table S5). Four morphological traits, tepal color, tepal nerve, tepal tip, and flower shape, were singly able to distinguish the two subgenera, *Eremurus* and *Henningia*, as defined by Wendelbo^15^ (Supplementary Table S6). In addition, tepal color, tepal tip and flower shape used in combination were able to differentiate the seven species. Overall, species were highly differentiated morphologically (*P* < 0.001); the morphological variability among species accounted for 70% of the total variability while the variability within species accounted for only 30% (Supplementary Table S7). The two most morphologically diverse species were *E. stenophyllus* and *E. spectabilis* with 10 and 19 morphotypes, and Shannon diversity indexes of 0.316 and 0.226, respectively (Table 2). *E. kopet-daghensis* was the least diverse with two morphotypes and a Shannon diversity index of 0.040.

**Table 2:** Summary of morphological diversity across species within the genus *Eremurus*

Species	Subgenus	Section	*N*	*H*′ (SE)	Morphotype	Private character	Diagnostic character
*E. spectabilis*	*Eremurus*	*Eremurus*	25	0.226 (0.078)	19	1	0
*E. inderiensis*	*Eremurus*	*Ammolirion*	6	0.186 (0.073)	5	1	0
*E. kopet-daghensis*	*Henningia*	*Henningia*	3	0.040 (0.040)	2	1	0
*E. luteus*	*Henningia*	*Henningia*	13	0.168 (0.061)	7	1	0
*E. stenophyllus*	*Henningia*	*Henningia*	13	0.316 (0.084)	10	0	3
*E. olgae*	*Henningia*	*Henningia*	17	0.125 (0.054)	8	0	0
*E. persicus*	*Henningia*	*Henningia*	8	0.071 (0.038)	4	0	0
Total			85	0.162 (0.025)	55	4	3

*N* no. of accessions, *H*′ Shannon-Weiner diversity index = −∑*p*_*i*_ ln(*p*_*i*_), *SE* standard error

Morphotype: number of different combinations of morphological characters; private character: character fixed in one species at a frequency of 100% and absent in all other species; diagnostic character: character present in one species at a frequency below 100% and absent in all other species

E_KERMANSHAH_39, E_S_62, and E_GH7 were not included in this analysis

*Eremurus spectabilis*, *E. inderiensis, E. kopet-daghensis*, and *E. luteus* each had one private morphological character, campanulate flower shape (*E. spectabilis*), tubular flower shape (*E. inderiensis*), pale pink tepals (*E. kopet-daghensis*), and pale yellow tepals (*E. luteus*). White, yellow, and orange tepal colors were diagnostic for *E. stenophyllus*. No variation was observed within species for tepal nerve, tepal tip, flower shape, bract margin, fruit shape, or leaf margin and surface indumentum. In contrast, inflorescence length displayed variation within all *Eremurus* species (Supplementary Table S6).

Overall, 55 morphotypes (matrices consisting of all 16 morphological characters) were recorded and all of them were specific to one of the studied species (Supplementary Table S8). Specific morphotypes were also recorded for each clade of *E. spectabilis, E. olgae, E. stenophyllus*, and *E. luteus* except *E. luteus* Clade II, where none of the five morphotypes observed were unique to Clade II (Supplementary Table S9). One trait out of the 16 evaluated (tepal color) was able to differentiate *E. stenophyllus* Clade I (yellow tepal color) from the rest of the *E. stenophyllus* accessions.

When accessions were color‐coded according to their genetic affiliation, the PCoA based on the 16 morphological traits showed a similar clustering of accessions to that obtained using the 3002 SNPs (Fig. 1d). The first coordinate of the PCoA explained 31.9% of the genetic variability and separated subgenus *Eremurus* from subgenus *Henningia*. Three traits, tepal color, tepal nerve, and tepal tip, were the main contributors (56%) to the variation explained by axis 1 (Supplementary Table S10). The second coordinate, explaining 23.3% of the genetic variability, distinguished *E. inderiensis* from *E. spectabilis* within subgenus *Eremurus*. The second coordinate also separated the species within subgenus *Henningia* into three groups represented by *E. olgae*, *E. stenophyllus* and the rest (*E. luteus, E. persicus, E. kopet-daghensis*) (Fig. 1d). The traits that were most highly correlated with axis 2 were stem length and leaf margin indumentum (35% contribution; Supplementary Table S10). The third coordinate explained 9.9% of the variation and distinguished *E. persicus* from *E. luteus* and *E. kopet-daghensis*. In addition, significant correlations between morphological and genetic distances across species were revealed (Rxy = 0.636, *P* = 0.010).

### Genetic analyses within genetic subpopulations in the genus *Eremurus*

#### SNP markers identified within *Eremurus* subpopulations

To resolve intraspecies relationships, GBS data for the three largest subpopulation groups identified by STRUCTURE at *K* = 5 and by phylogenetic analyses (*E. olgae/stenophyllus*, *E. spectabilis*, and *E. luteus*) were reanalyzed within each subpopulation to identify biallelic SNPs with a QD value ≥10, an allele frequency ≥10% and ≤15% missing data. Adjacent SNPs were also removed. A total of 22,934 reference tags were obtained for subpopulation ‘*spectabilis*’, 27,258 for subpopulation ‘*olgae/stenophyllus’* and 24,735 for subpopulation ‘*luteus’*. The highest percentage of unique reference tags was found in subpopulation ‘*spectabilis’* (65%), and the highest percentage of shared tags (23%) was observed between subpopulations ‘*olgae/stenophyllus’* and ‘*luteus’*. This concurs with *E. olgae, E. stenophyllus*, and *E. luteus* belonging to subgenus *Henningia*, and *E. spectabilis* belonging to subgenus *Eremurus*. Some 21% of the tags were shared between all three subpopulations (Supplementary Table S11). Using the generated GBS references in each subpopulation, we obtained 4175 SNPs for subpopulation *‘spectabilis’*, 5281 SNPs for subpopulation ‘*olgae/stenophyllus’* and 6131 SNPs for subpopulation *‘luteus’*. The majority (90.3%) of the SNP-carrying GBS reference tags were specific to a single subpopulation, 9.0% were common to two subpopulations, and 0.7% were shared between three subpopulations. The lower number of shared tags when considering only the SNP-carrying tags used in the analyses compared with all reference tags can be explained by the fact that common reference tags are not necessarily polymorphic in all subpopulations. The SNP markers, their location and the sequence of the corresponding GBS reads for subpopulations ‘*spectabilis*’, ‘*olgae/stenophyllus*’, and ‘*luteus*’ are given in Supplementary Tables S12, S13, and S14, respectively. The genotypic scores for each SNP marker for accessions within a subpopulation are given in Supplementary Tables S15, S16, and S17.

SNPs identified within subpopulations (‘subpopulation SNPs’) showed higher diversity indexes compared with SNPs identified across all species (‘species SNPs’) for each of the largest population groups investigated. Shannon’s information index *I* was 4.8-fold higher on average using ‘subpopulation SNPs’ (Table 3) than using ‘species SNPs’ (Table 1). Based on the ‘subpopulation SNPs’, *E. luteus* had the highest diversity among the four species analyzed (*E. luteus, E. olgae, E. stenophyllus*, and *E. spectabilis*) with a Shannon’s information index *I* of 0.632 (Table 3). It should be noted, however, that the ‘subpopulation SNPs’ were different for each species, except for *E. stenophyllus* and *E. olgae* where building of the GBS reference and SNP calling was done within the subpopulation *stenophyllus/olgae*. When using ‘species SNPs’, *E. luteus* presented the lowest genetic diversity (*I* = 0.088) of the four species (Table 1).

**Table 3:** Summary of diversity indexes and private SNPs in *Eremurus* species using SNPs identified within subpopulations (‘subpopulation SNPs’)

Species	SNP dataset	*N*	Ne (SE)	*I* (SE)	Ho (SE)	He (SE)	Fis (SE)	*P* (%)	Heterozygous SNPs (%)
*E. luteus*	6131	13	1.810 (0.002)	0.632 (0.001)	0.204 (0.002)	0.441 (0.001)	0.552 (0.003)	100.00	19.46
*E. stenophyllus*	5281	13	1.559 (0.004)	0.501 (0.002)	0.260 (0.002)	0.334 (0.002)	0.190 (0.005)	94.49	25.19
*E. olgae*		17	1.345 (0.005)	0.322 (0.004)	0.145 (0.002)	0.208 (0.003)	0.225 (0.005)	74.82	14.44
*E. spectabilis*	4175	26	1.701 (0.004)	0.587 (0.001)	0.179 (0.002)	0.401 (0.001)	0.587 (0.004)	100.00	16.96

*N* number of accessions, *Ne* number of effective alleles, *I* Shannon’s information index, *Ho* observed heterozygosity, *He* expected heterozygosity, *Fis* fixation index, *SE* standard error, *P* percentage of polymorphic loci (%)

SNP dataset: number of SNPs in the dataset used to calculate the diversity indexes; private SNPs: number of SNPs fixed in one species (at a frequency of 100%) and absent in all other species; diagnostic SNPs: number of SNPs present in one species at a frequency below 100% and absent in all other species

E_S_62 and E_GH7 were not included in this analysis

#### Phylogeographic analyses within STRUCTURE subpopulations

**Fig. 2:**
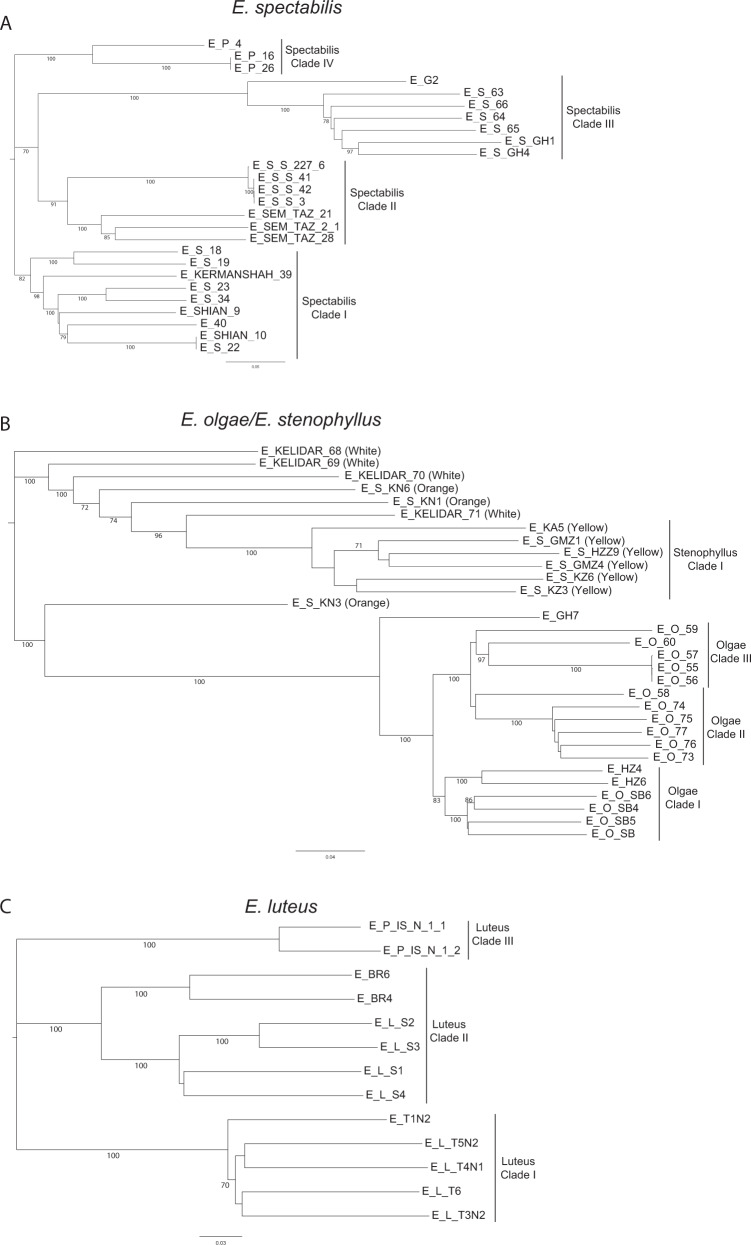
Phylogenetic trees within subpopulations. Trees within **a** *E. spectabilis*, **b** the *E. olgae/E. stenophyllus* species complex, and **c** *E. luteus* were generated using, respectively, 4175, 5281, and 6131 ‘subpopulation SNPs’. Bootstrap values for branches are indicated when higher than 70%. For accessions belonging to *E. stenophyllus*, flower color is given in parenthesis after the accession name

Using both larger SNP numbers and less conserved SNPs allowed most inter-accession relationships to be resolved with bootstrap values ≥70% (Fig. 2). Within subpopulation ‘*spectabilis*’ (Fig. 2a), four clades largely grouped accessions by geographic location. Clades I and IV were collected in the western part of Iran, while Clade II and Clade III were found in the center and eastern part of Iran, respectively (Fig. 1c).

As indicated by the phylogenetic trees obtained with both the ‘species SNPs’ and the ‘subpopulation SNPs’, subpopulation *‘olgae/stenophyllus*’ consisted of two sister clades, one comprising *E. olgae* accessions and the other comprising *E. stenophyllus* accessions (Figs. 1b and 2b). Both species were collected from the eastern part of Iran (Fig. 1c), and no geographic patterning at the regional level was observed that separated the two species. Discrepant placement in the two analyses was found for *E. stenophyllus* accession E_S_KN3, which was phylogenetically more closely related to *E. olgae* than to *E. stenophyllus* in the phylogeny using ‘subpopulation SNPs’, but grouped with *E. stenophyllus* in the across-species phylogeny and the STRUCTURE analysis at *K* = 6. An analysis of Nei’s genetic distance and genetic differentiation at the species level using ‘species SNPs’ showed that these two diversity indices were at least 3.6 (Nei’s distance) and 2.4-fold (Fst) lower between *E. stenophyllus* and *E. olgae* than between any other two species (Supplementary Table S4). *Eremurus stenophyllus* was the only species that had flower color variants. In addition to the typical yellow color, some accessions had orange or white flowers. All *E. stenophyllus* accessions with yellow-colored flowers grouped into a single clade I (Fig. 2b; Supplementary Table S9), but were not geographically isolated from the rest of the *E. stenophyllus* accessions. Within *E. olgae*, three clades were identified. While all *E. olgae* species were found in eastern Iran, some geographic patterning was found at the regional scale. Clade I was present at more western longitudes, while Clades II and III were prevalent at more eastern longitudes.

Three clades with unresolved relationships were identified in *E. luteus* (Fig. 2c). Clade I comprised five species collected at the same location (N36.3–E59.4) in eastern Iran. Clade II, also sampled in eastern Iran, consisted of two sister subclades, one comprising four species collected at N35.7–E61.1 and the other consisting of two species collected at N32.9–E59.2. Clade III comprised two accessions collected in the center of Iran (at N33.4–E53.9).

## Discussion

### Genotyping-by-sequencing for phylogenetic analysis across species within a genus

Genotyping-by-sequencing has been used in a number of species without a reference genome to identify SNP markers for genetic mapping or diversity analyses, e.g., refs. ^11^^,21–23^. Here, we demonstrate that the use of the methylation-sensitive restriction enzyme *Pst*I in combination with *Msp*I is effective even in species with a very large genome such as foxtail lily (1C = 8.1 Gb). Furthermore, using GBS references generated either across species or within species, the same GBS reads can be used to provide markers suitable for cross-species and intraspecific applications, respectively. To generate a reduced representation genome reference from GBS reads using the UGbS-Flex pipeline, we first clustered reads within accessions, extracted consensus sequences from each cluster, and then clustered the consensus sequences across accessions^11^. While we typically require a consensus sequence to be present in at least 50% of the accessions in order to be included in the reference, we did not apply this criterion for the generation of the cross-species reference. The main reason for not preselecting reference tags based on their prevalence in the set of samples was that the ‘ustacks’ program^24^ only groups sequences that fully overlap and we were concerned that the 50% threshold was too stringent, particularly because we did not know the level of divergence between the seven *Eremurus* species. We then used blast all-vs.-all to identify tags that had ≥98% homology and discarded all but one of the closely related sequences. This reference consisted of 201,099 sequences. Because we discarded SNPs with >30% of missing data, the 3002 SNPs used in the analysis were derived from highly conserved regions in the genome and were polymorphic at the species level rather than between accessions within a species. These 3002 SNPs clustered accessions by species with bootstrap values of ≥98% in NJ and UPGMA trees.

As expected, however, little bootstrap support was obtained for the majority of relationships between accessions within a species. To increase the resolution at the accession level, we extracted the raw reads for the three largest subpopulations obtained with STRUCTURE, which essentially corresponded to the species *E. stenophyllus/E. olgae*, *E. luteus*, and *E. spectabilis*. Generation of a GBS reference and SNP calling was then carried out within each subpopulation group. For this analysis, only sequences that were present in at least 50% of the accessions within a subpopulation were included in the reference, leading to smaller reference sets. Because of limitations on the number of SNPs that could be used within the phylogenetic program ‘DARwin’, we removed SNPs with an allele frequency <10% and >15% missing data, retaining 5281 SNPs for subpopulation ‘*olgae/stenophyllus’*, 6131 SNPs for subpopulation *‘luteus’* and 4175 SNPs for subpopulation *‘spectabilis’*.

### Genetic relationships between and within *Eremurus* species

We used STRUCTURE^20^, which applies a Bayesian iterative algorithm, to determine the most likely number of genetic groups and the membership of each *Eremurus* accession to these groups. We obtained five clusters (Fig. 1a), with no or very few admixed (≤90% membership to a single subpopulation) accessions within each cluster. E_K_49, E_K_52, and E_K_54, the only three *E. kopet-daghensis* accessions in our study, had ≥50% (but ≤90%) membership to subpopulation ‘*luteus*’ and minority membership (>10% and <50%) to the ‘*olgae/stenophyllus’* subpopulation. E_S_62, an accession identified based on morphological characters as *E. spectabilis*, had majority membership to *E. spectabilis* and minority membership to subpopulation ‘*olgae/stenophyllus*’. *E. olgae* and *E. stenophyllus* accessions fell within a single subpopulation. NJ and UPGMA analyses resolved the seven species into seven strongly supported clades. In agreement with the STRUCTURE results, the three *E. kopet-daghensis* accessions were sister to the *E. luteus* clade, and both clades were sister to the *E. olgae* and *E. stenophyllus* clades (Fig. 1b).

In 1876, Baker divided the genus *Eremurus* into three subgenera, *Eremurus verus, Ammolirion*, and *Henningia*^25^. Wendelbo^15^ recognized only two genera, *Eremurus*, which comprised sections *Eremurus* and *Ammolirion*, and *Henningia*, which comprised section *Henningia*. The seven *Eremurus* species found in Iran are distributed across the three genera/sections. *E. spectabilis* was classified as belonging to subgenus *Eremurus* section *Eremurus*, *E. inderiensis* as belonging to subgenus *Eremurus* section *Ammolirion*, and *E. luteus, E. olgae*, *E. persicus*, *E. stenophyllus*, and *E. kopet-daghensis* as belonging to subgenus *Henningia* section *Henningia*. Naderi Safar and colleagues^8^ subsequently showed using plastid *trn*L-F and ribosomal internal transcribed spacer (ITS) sequences that subgenus *Henningia* was paraphyletic, with *E. persicus* placed separately from the remainder of species belonging to this subgenus. Our results largely agree with Naderi Safar et al.^8^ with *E. luteus*, *E. kopet-daghensis*, *E. stenophyllus*, and *E. olgae* being located in one superclade (Superclade 1 in Supplementary Fig. S5), while *E. persicus* formed a separate superclade (Superclade 3 in Supplementary Fig. S5). Furthermore, pairwise Nei’s genetic distances showed that *E. persicus* was the most diverged of all *Eremurus* species analyzed (Supplementary Table S4), further supporting that *E. persicus* should be placed in a separate subgenus.

Our data also bring into question some of the current species delineations. Nei’s genetic difference and the genetic differentiation between any two species is, on average, 0.441 and 0.668, respectively. In contrast, these values are 0.059 and 0.217 when comparing *E. olgae* and *E. stenophyllus*. Furthermore, *E. olgae* and *E. stenophyllus* are inter-fertile, not geographically differentiated, and grouped in the same genetic subpopulation in a STRUCTURE analysis. We therefore recommend the use of ‘*olgae*’ and ‘*stenophyllus*’ at the subspecies level within the species complex *E. olgae/stenophyllus*.

### Phylogeography of *Eremurus* species in Iran

As expected, ‘subpopulation SNPs’ showed higher genetic diversity than ‘species SNPs’ suggesting that although ‘species SNPs’ are more efficient for species differentiation, ‘subpopulation SNPs’ are more accurate for diversity evaluation within species. The SNP data obtained from both the across-species and intraspecies analyses of the GBS reads demonstrated that accessions typically group by geographic location. Geographic distances and genetic distances calculated using ‘species SNPs’ were significantly correlated (Rxy = 0.439, *P* = 0.001) and the population of *Eremurus* accessions was geographically structured along a longitudinal axis. When Mantel tests were performed within species, the results revealed significant correlations between geographic and genetic distances (based on ‘subpopulation SNPs’) only for *E. persicus* (Rxy = 0.574, *P* = 0.020) and *E. spectabilis* (Rxy = 0.135, *P* = 0.05). Interestingly, *E. stenophyllus*, which typically has yellow flowers, was found in three color variants in the same geographic region (N36.72–E58.53). Yellow-colored accessions formed a single cluster, but the white and orange accessions did not cluster at the genetic level by flower color. This may not be too surprising considering that the color variants grow in sympatry and that foxtail lily is outcrossing.

### Levels of heterozygosity

When analyzing SNP variants across species (using ‘species SNPs’), the number of heterozygous loci identified within each accession was, on average, 3%. SNPs were called only for loci that had a sequencing depth of at least eight reads, which was sufficient to reliably identify heterozygous SNPs^11^. Hence, the paucity of heterozygous loci in foxtail lily, an outcrossing species, is not caused by a lack of read depth. Most likely, the SNP loci used for the across-species analyses were highly conserved and, consequently, alleles were fixed within a species. This is supported by the high correlation (*r*^2^ = 0.96, *P* < 0.001) between the overall diversity within a species and the percentage of heterozygous loci (Table 1). The only exception to the low occurrence of heterozygous SNPs was accession E_S_62, which had 31.1% heterozygotes and, based on STRUCTURE, NJ and PCoA analyses, was an interspecific hybrid between *E. spectabilis* and *E. olgae/stenophyllus*. However, no hybrids between species belonging to subgenus *Eremurus* and *Henningia* have been reported to date. Furthermore, E_S_62 had been identified morphologically as *E. spectabilis* and had the same morphotype as another *E. spectabilis* accession, E_S_64. Therefore, we deduce that the ‘hybrid’ status and high level of heterozygosity of E_S_62 were most likely caused by sample contamination.

When SNPs were used that were identified within subpopulations (‘subpopulation SNPs’), a higher percentage of heterozygous loci (on average, 19.5% for *E. luteus*, 17.0% for *E. spectabilis*, 14.4% for *E. olgae*, and 25.2% for *E. stenophyllus*) were identified (Table 3), commensurate with *Eremurus* species being predominantly outcrossing.

### Morphological characterization

Of the 16 evaluated traits, tepal color was the most variable trait and was informative for subgenus differentiation according to Wendelbo^15^. *Eremurus persicus*, which should be placed in its own subgenus based on the genetic data, could be distinguished from other species in Wendelbo’s subgenus *Henningia* by a hairy leaf surface. Although only four species of the genus *Eremurus* (*E. spectabilis, E. inderiensis, E. kopet-daghensis*, and *E. luteus)* displayed private morphological characters, a set of three morphological traits (flower shape, tepal color, and tepal tip) was sufficient to differentiate the seven species, highlighting the importance of morphological characterization. Species clustering based on morphological data in the PCoA was driven largely by the few flower characteristics that are key identifiers for *Eremurus* species. Most vegetative characters contributed little to the PCoA axes. Consequently, species classification based on morphology could only be done unambiguously at the flowering stage. When only the eight vegetative traits measured in our study were considered, some species’ morphotypes overlapped. Similar morphotypes were seen not only within subgenera, but also across subgenera, indicating that the eight vegetative traits are insufficient to differentiate accessions at the subgenus level. In contrast, all species could be identified at any stage during their life cycle using a panel of seven SNPs. Any SNP with species-specific (or private) alleles (indicated in Supplementary Table S2) could be used singly to unambiguously identify that species or, in the case of *E. stenophyllus/E. olgae*, the species complex. No private alleles were identified that uniquely identified *E. stenophyllus* or *E. olgae*. However, three markers diagnostic for *E. stenophyllus* (M0087, M0367, and M0368) each could distinguish all 17 *E. stenophyllus* accessions analyzed from the 13 *E. olgae* accessions. With the exception of three *E. stenophyllus* accessions that were heterozygous, all *E. stenophyllus* accessions were homozygous for the alternate allele, while *E. olgae* accessions were homozygous for the reference allele. Although morphological and genetic distances were highly correlated, the genetic markers presented in this work definitely represent the most accurate and rapid method to resolve species and subspecies classifications of accessions within the genus *Eremurus*, in particular during the vegetative growth stage. For example, E_GH7 and E_KERMANSHAH_39, two accessions that were collected at the vegetative stage and classified only at the genus level were identified as *E. olgae* and *E. spectabilis*, respectively, using SNP markers. Furthermore, the SNP markers with private (species-specific) alleles provide a rapid method for phenotyping of hybrids.

### Conclusions

Our study provides the first use of GBS in an angiosperm species with a haploid genome size larger than 8 Gb. Despite the absence of a reference genome, SNPs were successfully identified across species within the genus *Eremurus* as well as within *Eremurus* species using GBS reference tags that were assembled across all species (‘species SNPs’) or within species/subpopulations (‘subpopulation SNPs’), respectively. Our data demonstrated longitudinal geographic stratification at the country level for the genus and for the species *E. spectabilis* and *E. luteus* and, at the regional scale, for *E. olgae*. While classification of species based on morphology was robust, the SNPs provided a tool to identify species during the vegetative stage, which should be particularly useful for breeding purposes, including identification of diverse parents for crossing, hybrid identification, and cultivar protection. Furthermore, the SNPs provided important new information regarding the genetic relatedness of species within the genus *Eremurus* that suggests that reclassification at the subgenus and species level is in order.

## Materials and methods

### Sample collection

Leaves were collected from wild *Eremurus* populations in Iran during the spring and early summer of 2015 and 2016, and stored at −20 °C until further use. A total of 143 genotypes belonging to seven species were collected from nine provinces. One to six individuals were sampled per location. The majority of species were identified in situ based on flower morphology. For each accessions, 16 morphological characteristics were measured (inflorescence length, stem length, leaf length, leaf number, stem diameter, rhizome number, rhizome diameter, peduncle length, tepal color, tepal nerve, tepal tip, flower shape, bract margin, fruit shape, margin of leaves indumentum, and surface of leaves indumentum) which, combined, defined an accession’s morphotype. The subset of 88 genotypes that was successfully analyzed by GBS, together with their species designation based on morphological characteristics and genetic data, subgenus, geographic origin and morphotype, is presented in Supplementary Table S1. Source locations are shown in Supplementary Fig. S1.

### DNA extraction and genotyping

Genomic DNA was isolated from frozen leaf tissue using a CTAB procedure^26^. The DNA quantity and quality were determined by Nanodrop spectrophotometry (Thermo Scientific) and agarose gel electrophoresis. Ninety-six *Eremurus spp*. samples that had high DNA quality and were representative of the sampled populations were chosen for GBS analysis. GBS was done as described by Qi et al.^11^ using the enzyme combination *Pst*I/*Msp*I. Briefly, 250 ng of DNA from each sample was double-digested with *Pst*I and *Msp*I, and ligated to a barcoded adapter at the *Pst*I site and a common Y-adapter at the *Msp*I site. Unligated adaptors were removed with OMEGA Mag-bind RXNPure plus Beads. Samples were PCR-amplified separately and the individual libraries were quantified using SYBR Green. Amplicon size range for 11 samples from the high and low end of the range were verified on a Bioanalyzer (Agilent). An epMotion 5075 pipetting system was used to pool 5 ng of each of the 96 samples. The pooled library sample was quantified by Qubit and a subsample was run on a fragment analyzer. A KAPA Library Quantification Kit was used to determine library concentration prior to sequencing on a NextSeq (150 cycles) SE 150 Mid Output flow cell.

### Generation of a GBS reference and SNP calling

Processing of the GBS reads and generation of a GBS reference using the scripts ‘ustacks’^24^ and ‘ASustacks’^11^ were essentially done as described in Qi et al.^11^. For interspecific analyses, the GBS reference was generated across all accessions within the genus *Eremurus*. For intraspecific analyses, the GBS reference was generated across accessions within a species. The parameters used in ‘ustacks’ and ‘ASustacks’ were ‘-m 2, -M 2 and –N 4’. Tags that were present in at least two accessions and at least 50% of the accessions were included in the inter- and intraspecific GBS references, respectively. If two or more tags had ≥98% sequence identity, only a single tag was included in the reference^11^.

Reads from each accession were aligned to the relevant GBS reference(s) with Bowtie 2^27^, and SNP calling was done using Unified Genotyper from the Genome Analysis Toolkit (GATK)^28^ using the work flow and scripts described in Qi et al.^11^. SNP filtering included removal of SNPs with three or more alleles, removal of SNPs with allele frequencies <0.1 and >0.9, and removal of adjacent SNPs. SNPs with a read depth of at least 8X were converted to the mapping scores A, B, H, D (A or H), and C (B or H)^11^. These scores were later converted for use in GenAlEx to the format 11 (A), 22 (B), and 12 (H); C and D scores were changed to missing data points (00). Markers with more than 50% of missing data were removed.

### Identification of GBS reference tags shared between species

Intraspecific GBS references were generated for each of the three largest subpopulation groups as determined by STRUCTURE (see below). To identify GBS reference tags that were shared between the three subpopulations analyzed, the reference tags belonging to each subpopulation were pooled and compared with one another using BLASTN. If two or more tags had ≥95% sequence identity, only a single tag was kept. All tags with <95% sequence identity across the three subpopulations formed the non-redundant tag set. GBS reference tags from each population were then compared with the non-redundant tag set using BLASTN to identify tags that were unique to that population or shared between populations.

### Population structure analysis

The population structure of the genotyped *Eremurus spp*. germplasm was determined based on the SNP set identified across all 88 genotyped accessions belonging to seven Iranian *Eremurus* species. Genetic subpopulations were identified using the Bayesian clustering procedure implemented in STRUCTURE v.2.3.4^20^ with ten runs of the admixture model, a burn-in period of 100,000 replications, a run length of 100,000 Markov Chain Monte Carlo (MCMC) iterations and the number of putative subpopulations (*K*) ranging from one to ten. The optimum value of *K* was selected based on the Delta *K* estimate of Evanno et al.^29^ using Structure Harvester^19^. Accessions with a membership probability to a single subpopulation larger than 90% were considered genetically pure. Accessions with membership ≤90% to a single subpopulation were considered admixed. A principal coordinates analysis (PCoA) was performed using the same dataset with GenAlEx 6.502^30^.

### Genetic diversity and phylogenetic analysis based on SNP markers

The number of effective alleles (Ne), number of private SNPs, percentage of polymorphic loci (*P*), Shannon’s information index (*I*), observed and expected heterozygosity (Ho, He) and fixation index (Fis) were calculated using GenAlEx 6.502^30^ and values were compared across the seven species. The overall genetic distance (Fst) and estimated gene flow (*N*_m_) between species were also calculated in GenAlEx 6.502^30^. In addition, the correlation between genetic and geographic distance was analyzed across all accessions as well as within species using a Mantel test implemented in GenAlEx 6.502. Phylogenetic analyses using the Neighbor Joining (NJ) and Unweighted Pair Group Method with Arithmetic mean (UPGMA) methods, and a bootstrap test with 500 replications were performed with DARwin 6.0.14 software^31^ to reveal relationships within the genus *Eremurus*. In addition, the pairwise Nei’s genetic distance and Fst genetic differentiation were calculated between the seven species of the *Eremurus* genus using SNPs identified across species (‘species SNPs’) and between clades within the largest population groups using SNPs identified within subpopulations (‘subpopulation SNPs’) in GenAlEx 6.502.

To examine the power of the SNP markers to detect unique multilocus genotypes (MLGs), we generated genotype accumulation curves using the total ‘species SNP’ dataset (3002 SNPs) and random subsets of 100, 200, 500, and 1000 SNPs using the function ‘genotype-curve’ implemented in the R3.2.2^32^ package ‘ppopr’. The genotype-curve function randomly samples different subsets of SNPs without replacement and plots the relationship between the number of SNPs scored and the number of MLGs identified.

### Genetic diversity based on morphological characteristics

The 16 morphological traits scored for species identification were compared for their Shannon-Weiner diversity index (*H*′) using the following formula: *H*′ = −∑*p*_*i*_ ln(*p*_*i*_), where *p*_*i*_ is the frequency of the *i*th character. The morphological diversity of each species was estimated by calculating the *H*′ diversity index, the number of morphotypes (number of different combinations of morphological characters), the number of private morphotypes (number of morphotypes present in a single species only), the number of private characters (characters fixed in one species at a frequency of 100% and absent in all other species) and diagnostic characters (characters present in one species at a frequency below 100% and absent in all other species). The number of total morphotypes and private morphotypes were also calculated by clade for the three largest subpopulation groups as determined by STRUCTURE. In addition, morphological differentiation within the genus *Eremurus* was investigated based on the 16 traits by a Principal Coordinates Analysis (PCoA) using GenAlEx 6.502^30^. The contribution of each morphological trait to axes 1 and 2 was calculated using the R3.2.2^32^ packages ‘FactoMineR’ and ‘Factoextra’. Variability among species and within species for morphological traits was assessed in GenAlEx 6.502^30^. Finally, a Mantel test was performed between the genetic and morphological matrix distances across the genotyped accessions using GenAlEx 6.502.

## Supplementary information

Supplementary tables

Figure S1

Figure S2

Figure S3

Figure S4

Figure S5

## Data Availability

Raw GBS reads have been submitted to NCBI’s short read archive (SRA) (PRJNA544250). All SNPs used in the analyses, together with flanking sequence, and the genotypic scores for all accessions have been included as supplementary information. All other relevant information is included as tables, figures, or supplementary information.
