# Supplementary material for: Intra- and interspecific diversity analyses in the genus Eremurus in Iran using genotyping-by-sequencing reveal geographic population structure
Source: Hortic Res. 2020 Mar 2;7:30. doi: 10.1038/s41438-020-0265-9 (PMC7052146; doi:10.1038/s41438-020-0265-9)
Supplement: Supplementary file 2 — Figure S1 [file 41438_2020_265_MOESM2_ESM.pptx]

## Slide 1
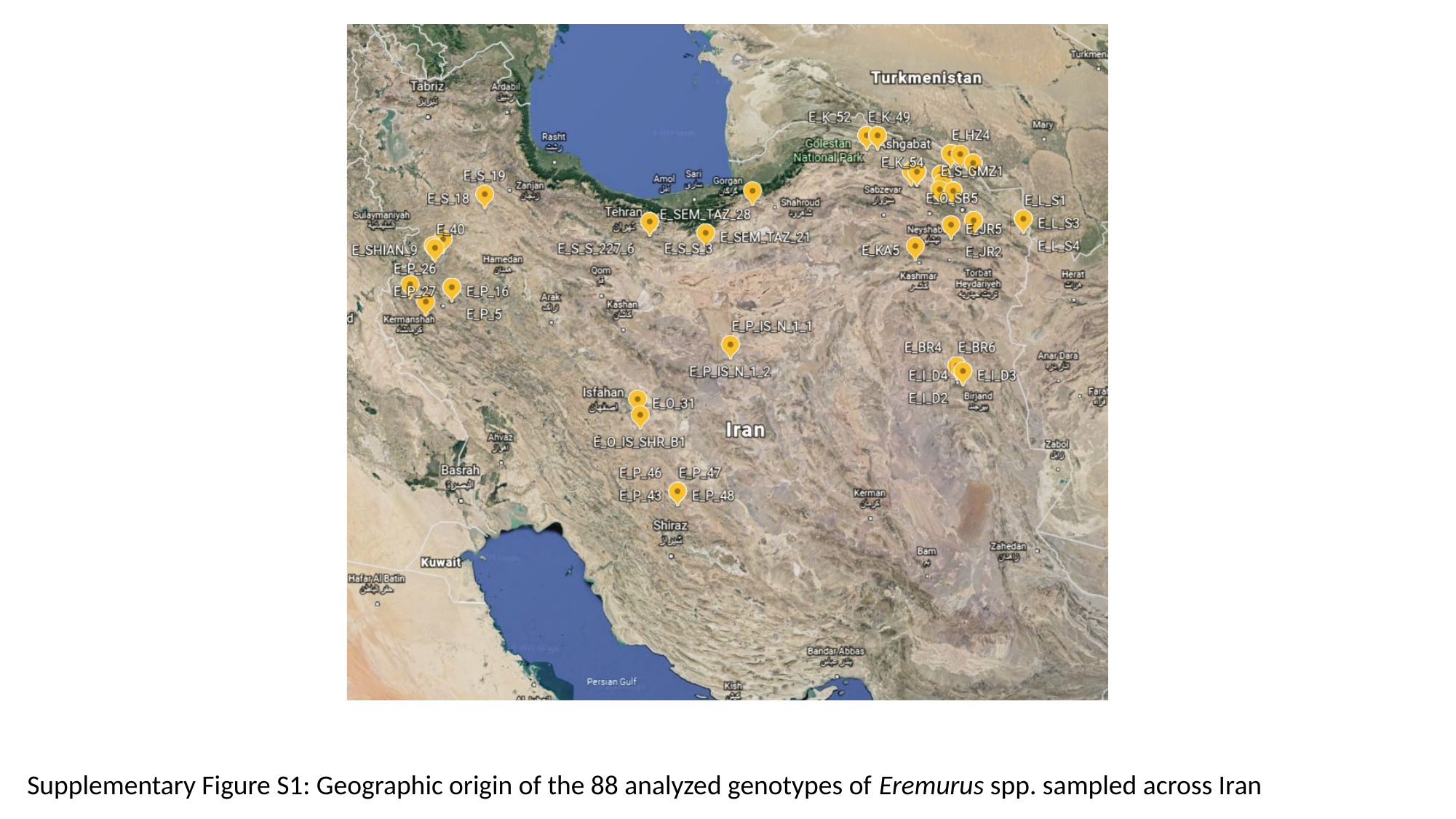

Supplementary Figure S1: Geographic origin of the 88 analyzed genotypes of Eremurus spp. sampled across Iran
