# Supplementary material for: Intra- and interspecific diversity analyses in the genus Eremurus in Iran using genotyping-by-sequencing reveal geographic population structure
Source: Hortic Res. 2020 Mar 2;7:30. doi: 10.1038/s41438-020-0265-9 (PMC7052146; doi:10.1038/s41438-020-0265-9)
Supplement: Supplementary file 3 — Figure S2 [file 41438_2020_265_MOESM3_ESM.pptx]

## Slide 1
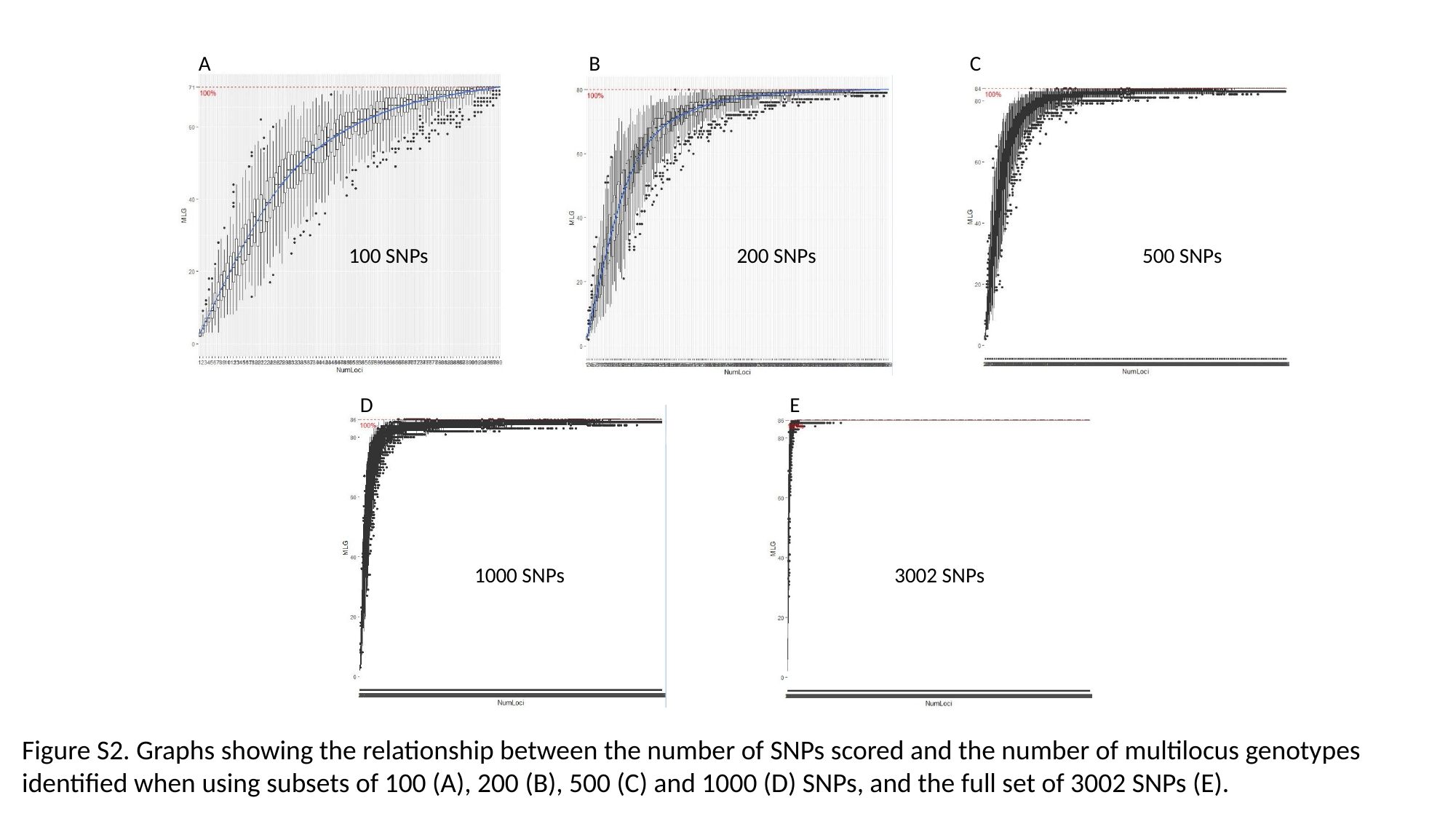

A
B
C
100 SNPs
200 SNPs
500 SNPs
D
E
1000 SNPs
3002 SNPs
Figure S2. Graphs showing the relationship between the number of SNPs scored and the number of multilocus genotypes identified when using subsets of 100 (A), 200 (B), 500 (C) and 1000 (D) SNPs, and the full set of 3002 SNPs (E).
