# Supplementary material for: Intra- and interspecific diversity analyses in the genus Eremurus in Iran using genotyping-by-sequencing reveal geographic population structure
Source: Hortic Res. 2020 Mar 2;7:30. doi: 10.1038/s41438-020-0265-9 (PMC7052146; doi:10.1038/s41438-020-0265-9)
Supplement: Supplementary file 5 — Figure S4 [file 41438_2020_265_MOESM5_ESM.pptx]

## Slide 1
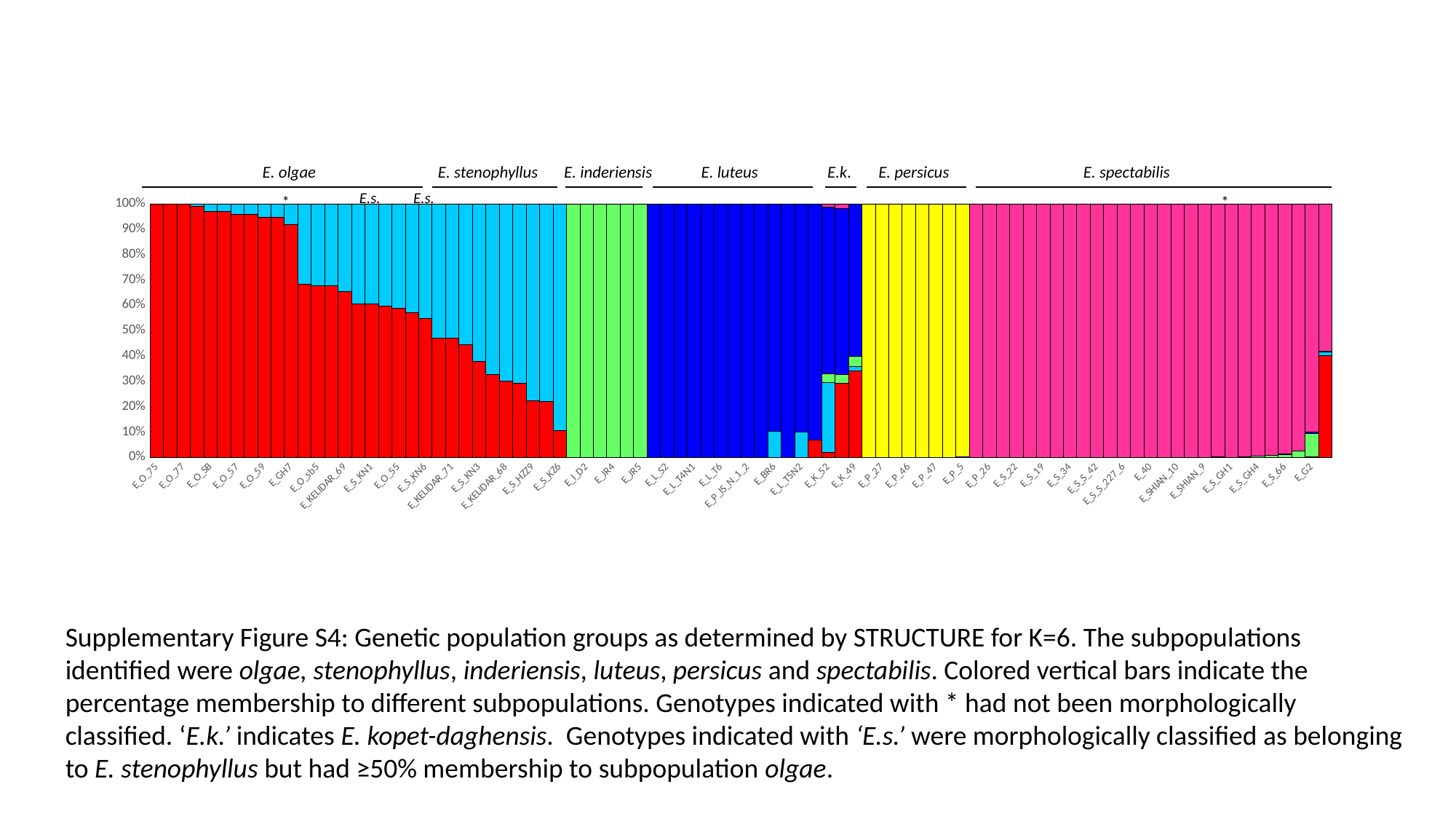

E. olgae
E. stenophyllus
E. inderiensis
E. luteus
E.k.
E. persicus
E. spectabilis
E.s.
E.s.
*
*
### Chart
| Category | | | | | | |
|---|---|---|---|---|---|---|
| E_O_75 | 0.999 | 0.0 | 0.0 | 0.0 | 0.0 | 0.0 |
| E_O_76 | 0.999 | 0.0 | 0.0 | 0.0 | 0.0 | 0.0 |
| E_O_77 | 0.999 | 0.0 | 0.0 | 0.0 | 0.0 | 0.0 |
| E_O_73 | 0.99 | 0.01 | 0.0 | 0.0 | 0.0 | 0.0 |
| E_O_SB | 0.971 | 0.029 | 0.0 | 0.0 | 0.0 | 0.0 |
| E_O_SB4 | 0.969 | 0.031 | 0.0 | 0.0 | 0.0 | 0.0 |
| E_O_57 | 0.959 | 0.041 | 0.0 | 0.0 | 0.0 | 0.0 |
| E_O_56 | 0.959 | 0.04 | 0.0 | 0.0 | 0.0 | 0.0 |
| E_O_59 | 0.947 | 0.053 | 0.0 | 0.001 | 0.0 | 0.0 |
| E_O_58 | 0.946 | 0.054 | 0.0 | 0.0 | 0.0 | 0.0 |
| E_GH7 | 0.917 | 0.083 | 0.0 | 0.0 | 0.0 | 0.0 |
| E_HZ6 | 0.683 | 0.316 | 0.0 | 0.0 | 0.0 | 0.0 |
| E_O_sb5 | 0.678 | 0.322 | 0.0 | 0.0 | 0.0 | 0.0 |
| E_HZ4 | 0.678 | 0.321 | 0.0 | 0.001 | 0.0 | 0.0 |
| E_KELIDAR_69 | 0.654 | 0.346 | 0.0 | 0.0 | 0.0 | 0.0 |
| E_O_74 | 0.606 | 0.394 | 0.0 | 0.0 | 0.0 | 0.0 |
| E_S_KN1 | 0.604 | 0.396 | 0.0 | 0.0 | 0.0 | 0.0 |
| E_O_SB6 | 0.596 | 0.403 | 0.0 | 0.0 | 0.0 | 0.0 |
| E_O_55 | 0.589 | 0.411 | 0.0 | 0.0 | 0.0 | 0.0 |
| E_O_60 | 0.571 | 0.428 | 0.0 | 0.0 | 0.0 | 0.0 |
| E_S_KN6 | 0.548 | 0.452 | 0.0 | 0.0 | 0.0 | 0.0 |
| E_S_KZ3 | 0.471 | 0.528 | 0.0 | 0.0 | 0.0 | 0.0 |
| E_KELIDAR_71 | 0.469 | 0.529 | 0.0 | 0.001 | 0.0 | 0.001 |
| E_KA5 | 0.445 | 0.554 | 0.0 | 0.0 | 0.0 | 0.0 |
| E_S_KN3 | 0.379 | 0.621 | 0.0 | 0.0 | 0.0 | 0.0 |
| E_KELIDAR_70 | 0.328 | 0.672 | 0.0 | 0.0 | 0.0 | 0.0 |
| E_KELIDAR_68 | 0.301 | 0.699 | 0.0 | 0.0 | 0.0 | 0.0 |
| E_S_GMZ1 | 0.292 | 0.708 | 0.0 | 0.0 | 0.0 | 0.0 |
| E_S_HZZ9 | 0.223 | 0.775 | 0.0 | 0.002 | 0.0 | 0.0 |
| E_S_GMZ4 | 0.22 | 0.78 | 0.0 | 0.0 | 0.0 | 0.0 |
| E_S_KZ6 | 0.105 | 0.895 | 0.0 | 0.0 | 0.0 | 0.0 |
| E_I_D4 | 0.0 | 0.0 | 1.0 | 0.0 | 0.0 | 0.0 |
| E_I_D2 | 0.0 | 0.0 | 1.0 | 0.0 | 0.0 | 0.0 |
| E_I_D3 | 0.0 | 0.0 | 1.0 | 0.0 | 0.0 | 0.0 |
| E_JR4 | 0.0 | 0.0 | 1.0 | 0.0 | 0.0 | 0.0 |
| E_JR2 | 0.0 | 0.0 | 1.0 | 0.0 | 0.0 | 0.0 |
| E_JR5 | 0.0 | 0.0 | 1.0 | 0.0 | 0.0 | 0.0 |
| E_L_S4 | 0.0 | 0.0 | 0.0 | 1.0 | 0.0 | 0.0 |
| E_L_S2 | 0.0 | 0.0 | 0.0 | 1.0 | 0.0 | 0.0 |
| E_L_S1 | 0.0 | 0.0 | 0.0 | 1.0 | 0.0 | 0.0 |
| E_L_T4N1 | 0.0 | 0.0 | 0.0 | 1.0 | 0.0 | 0.0 |
| E_L_T3N2 | 0.0 | 0.0 | 0.0 | 1.0 | 0.0 | 0.0 |
| E_L_T6 | 0.0 | 0.0 | 0.0 | 1.0 | 0.0 | 0.0 |
| E_T1N2 | 0.0 | 0.0 | 0.0 | 1.0 | 0.0 | 0.0 |
| E_P_IS_N_1_2 | 0.0 | 0.0 | 0.0 | 1.0 | 0.0 | 0.0 |
| E_P_IS_N_1_1 | 0.0 | 0.0 | 0.0 | 1.0 | 0.0 | 0.0 |
| E_BR6 | 0.0 | 0.103 | 0.0 | 0.897 | 0.0 | 0.0 |
| E_BR4 | 0.0 | 0.0 | 0.0 | 0.999 | 0.0 | 0.001 |
| E_L_T5N2 | 0.0 | 0.101 | 0.0 | 0.899 | 0.0 | 0.0 |
| E_L_S3 | 0.067 | 0.0 | 0.0 | 0.933 | 0.0 | 0.0 |
| E_K_52 | 0.018 | 0.278 | 0.034 | 0.656 | 0.0 | 0.014 |
| E_K_54 | 0.292 | 0.001 | 0.035 | 0.653 | 0.0 | 0.019 |
| E_K_49 | 0.342 | 0.015 | 0.041 | 0.601 | 0.0 | 0.001 |
| E_O_31 | 0.0 | 0.0 | 0.0 | 0.0 | 1.0 | 0.0 |
| E_P_27 | 0.0 | 0.0 | 0.0 | 0.0 | 1.0 | 0.0 |
| E_P_48 | 0.0 | 0.0 | 0.0 | 0.0 | 1.0 | 0.0 |
| E_P_46 | 0.0 | 0.0 | 0.0 | 0.0 | 1.0 | 0.0 |
| E_P_43 | 0.0 | 0.0 | 0.0 | 0.0 | 1.0 | 0.0 |
| E_P_47 | 0.0 | 0.0 | 0.0 | 0.0 | 1.0 | 0.0 |
| E_O_IS_SHR_B1 | 0.0 | 0.0 | 0.0 | 0.0 | 0.999 | 0.0 |
| E_P_5 | 0.001 | 0.0 | 0.0 | 0.001 | 0.999 | 0.0 |
| E_P_16 | 0.0 | 0.0 | 0.0 | 0.0 | 0.0 | 1.0 |
| E_P_26 | 0.0 | 0.0 | 0.0 | 0.0 | 0.0 | 1.0 |
| E_P_4 | 0.0 | 0.0 | 0.0 | 0.0 | 0.0 | 0.999 |
| E_S_22 | 0.0 | 0.0 | 0.0 | 0.0 | 0.0 | 1.0 |
| E_S_18 | 0.0 | 0.0 | 0.0 | 0.0 | 0.0 | 1.0 |
| E_S_19 | 0.0 | 0.0 | 0.0 | 0.0 | 0.0 | 0.999 |
| E_SEM_TAZ_2_1 | 0.0 | 0.0 | 0.0 | 0.0 | 0.0 | 1.0 |
| E_S_34 | 0.0 | 0.0 | 0.0 | 0.0 | 0.0 | 1.0 |
| E_S_23 | 0.0 | 0.0 | 0.0 | 0.0 | 0.0 | 1.0 |
| E_S_S_42 | 0.0 | 0.0 | 0.0 | 0.0 | 0.0 | 1.0 |
| E_S_S_3 | 0.0 | 0.0 | 0.0 | 0.0 | 0.0 | 1.0 |
| E_S_S_227_6 | 0.0 | 0.0 | 0.0 | 0.0 | 0.0 | 1.0 |
| E_S_S_41 | 0.0 | 0.0 | 0.0 | 0.0 | 0.0 | 1.0 |
| E_40 | 0.0 | 0.0 | 0.0 | 0.0 | 0.0 | 1.0 |
| E_SEM_TAZ_21 | 0.0 | 0.0 | 0.0 | 0.0 | 0.0 | 1.0 |
| E_SHIAN_10 | 0.0 | 0.0 | 0.0 | 0.0 | 0.0 | 1.0 |
| E_SEM_TAZ_28 | 0.0 | 0.0 | 0.0 | 0.0 | 0.0 | 1.0 |
| E_SHIAN_9 | 0.0 | 0.0 | 0.0 | 0.0 | 0.0 | 1.0 |
| E_KERMANSHAH_39 | 0.0 | 0.0 | 0.001 | 0.0 | 0.0 | 0.999 |
| E_S_GH1 | 0.0 | 0.0 | 0.0 | 0.0 | 0.0 | 0.999 |
| E_S_63 | 0.0 | 0.0 | 0.003 | 0.0 | 0.0 | 0.997 |
| E_S_GH4 | 0.0 | 0.0 | 0.005 | 0.0 | 0.0 | 0.995 |
| E_S_64 | 0.0 | 0.0 | 0.009 | 0.0 | 0.0 | 0.991 |
| E_S_66 | 0.0 | 0.0 | 0.01 | 0.003 | 0.0 | 0.986 |
| E_S_65 | 0.0 | 0.0 | 0.024 | 0.0 | 0.0 | 0.975 |
| E_G2 | 0.0 | 0.002 | 0.093 | 0.004 | 0.0 | 0.901 |
| E_S_62 | 0.401 | 0.016 | 0.0 | 0.002 | 0.0 | 0.582 |Supplementary Figure S4: Genetic population groups as determined by STRUCTURE for K=6. The subpopulations identified were olgae, stenophyllus, inderiensis, luteus, persicus and spectabilis. Colored vertical bars indicate the percentage membership to different subpopulations. Genotypes indicated with * had not been morphologically classified. ‘E.k.’ indicates E. kopet-daghensis. Genotypes indicated with ‘E.s.’ were morphologically classified as belonging to E. stenophyllus but had ≥50% membership to subpopulation olgae.
