# Supplementary material for: Intra- and interspecific diversity analyses in the genus Eremurus in Iran using genotyping-by-sequencing reveal geographic population structure
Source: Hortic Res. 2020 Mar 2;7:30. doi: 10.1038/s41438-020-0265-9 (PMC7052146; doi:10.1038/s41438-020-0265-9)
Supplement: Supplementary file 6 — Figure S5 [file 41438_2020_265_MOESM6_ESM.pdf]

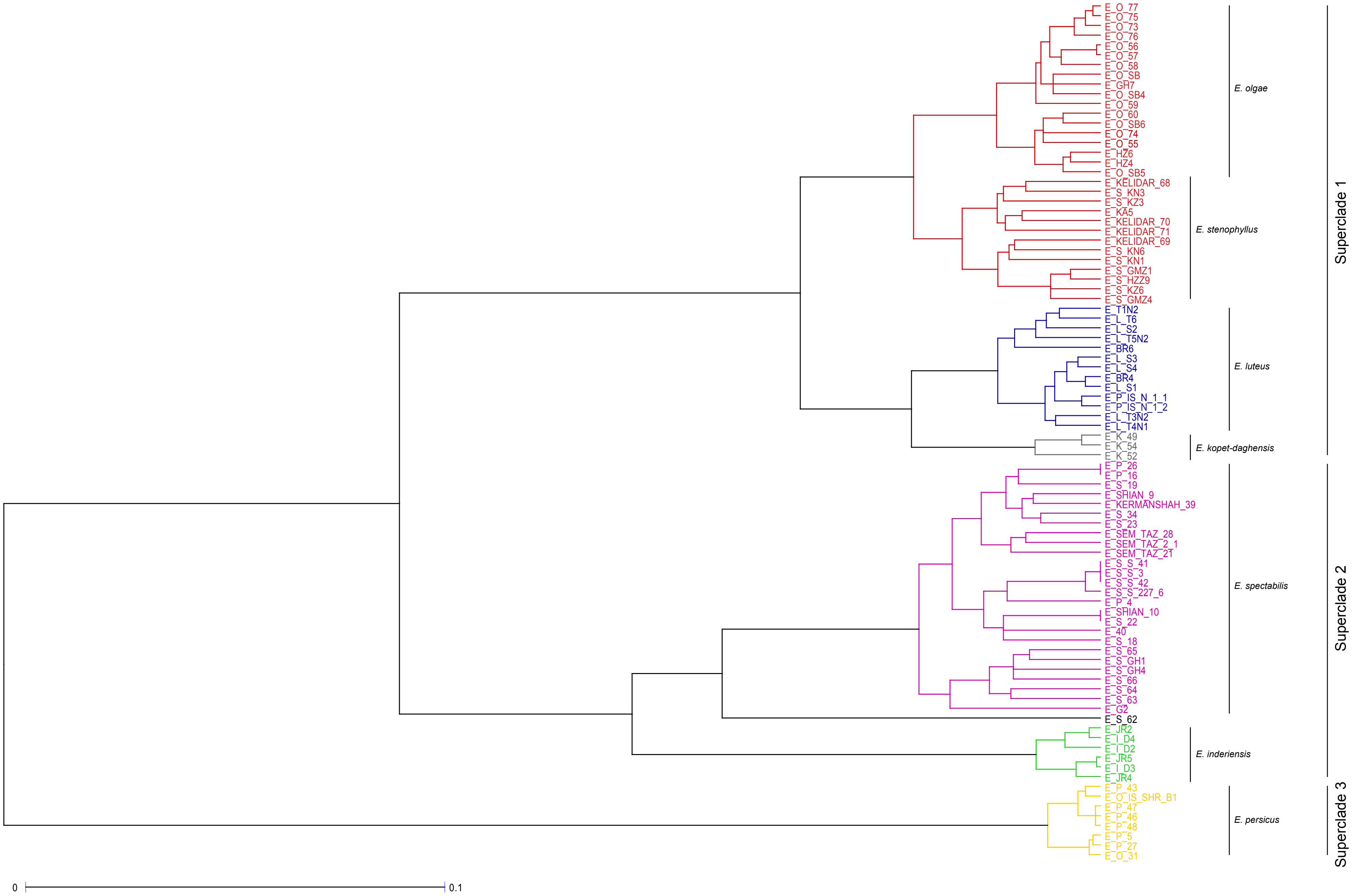

Figure S5: UPGMA tree based on 3002 ‘species SNPs’ showing the relationships between seven *Eremurus* species.
